# Health‐related quality of life and health status in persons with haemophilia A with inhibitors: A prospective, multicentre, non‐interventional study (NIS)

**DOI:** 10.1111/hae.13731

**Published:** 2019-04-24

**Authors:** Johnny Mahlangu, Johannes Oldenburg, Michael U. Callaghan, Midori Shima, Maria Elisa Mancuso, Peter Trask, Michael Recht, Claudia Garcia, Renchi Yang, Michaela Lehle, Harrison Macharia, Elina Asikanius, Gallia G. Levy, Rebecca Kruse‐Jarres, Sylvia von Mackensen

**Affiliations:** ^1^ Haemophilia Comprehensive Care Centre, Faculty of Health Sciences University of the Witwatersrand and NHLS Johannesburg South Africa; ^2^ Department of Experimental Haematology and Transfusion Medicine University Clinic Bonn Bonn Germany; ^3^ Children's Hospital of Michigan Detroit Michigan; ^4^ Department of Pediatrics Nara Medical University Kashihara, Nara Japan; ^5^ Fondazione IRCCS Ca' Granda, Ospedale Maggiore Policlinico Milan Italy; ^6^ Genentech, Inc. South San Francisco California; ^7^ Oregon Health & Science University Portland Oregon; ^8^ Haematology Department Hospital Mexico San Jose Costa Rica; ^9^ Institute of Hematology and Blood Diseases Hospital Chinese Academy of Medical Sciences Tianjin China; ^10^ F. Hoffmann‐La Roche Ltd Basel Switzerland; ^11^ Bloodworks Northwest Seattle Washington; ^12^ University Medical Centre Hamburg‐Eppendorf Hamburg Germany

**Keywords:** alloantibodies, haemophilia, health‐related quality of life, inhibitors, non‐interventional

## Abstract

**Introduction:**

Real‐world data (RWD) on health‐related outcomes in persons with haemophilia A (PwHA) provide insights into patient needs and can guide clinical study design. A global, prospective, non‐interventional study (NIS; NCT02476942) collected detailed RWD on bleeding outcomes, health‐related quality of life (HRQoL) and health status in PwHA treated per local routine clinical practice.

**Aim:**

To report HRQoL and health status in the adult/adolescent PwHA with inhibitors cohort in the NIS.

**Methods:**

This cohort enrolled PwHA aged ≥12 years with high‐titre factor VIII inhibitor history. Participants remained on their usual treatment (no protocol‐specified interventions). Health‐related outcomes: Haemophilia Quality of Life Questionnaire for Adults (Haem‐A‐QoL), Haemophilia‐specific Quality of Life Questionnaire for Children Short Form (Haemo‐QoL SF), EuroQol 5‐Dimensions 5‐Levels (EQ‐5D‐5L) index utility score (IUS) and visual analogue scale (EQ‐VAS).

**Results:**

One hundred three participants were enrolled on episodic (n = 75) or prophylactic treatment (n = 28); median (range) age, 31 (12‐75) years; median (range) observation time, 26 (4‐70) weeks. Haem‐A‐QoL scores indicated impairments in HRQoL aspects; comparable between episodic/prophylactic regimens and relatively consistent over time. Haemo‐QoL SF scores with both regimens varied over time, and appeared poorer with episodic than prophylactic treatment. IUS and EQ‐VAS were comparable between regimens, stable over time and lower on bleeding days. Mean proportions of missed work and school days were 16% and 23%, respectively; mean (standard deviation) number of days hospitalized was 3.2 (8.8) (comparable between groups).

**Conclusions:**

These RWD demonstrate that PwHA with inhibitors have impaired HRQoL, despite standard treatment, and that more effective treatment options are needed.

## 1 INTRODUCTION

Haemophilia A, characterized by coagulation factor VIII (FVIII) deficiency, is the most prevalent form of haemophilia.^1^ Persons with haemophilia A (PwHA) are at high risk of frequent and prolonged bleeding^2^ and related sequelae. This may lead to poor quality of life and can affect emotional, social and physical components of patients' well‐being and function.^3^, ^4^, ^5^, ^6^ The current standard of care for PwHA is intravenous FVIII replacement therapy, which leads to the development of anti‐FVIII alloantibodies (inhibitors) in up to 30% of previously untreated PwHA,^7^ reducing treatment effects, limiting treatment options and leading to increased risk of morbidity and mortality.^8^, ^9^

Standard therapeutic options for PwHA with inhibitors include immune tolerance induction (ITI), which attempts to eradicate inhibitors, and bypassing agents (BPAs) to prevent or treat bleeding.^10^ Prophylaxis with BPAs is especially burdensome, requiring infusions every other day.^11^, ^12^ Consequently, the majority of PwHA with inhibitors receive episodic BPA treatment.^13^ The efficacy of BPAs in the treatment or prevention of bleeding has been shown to be suboptimal.^8^, ^14^, ^15^

Validated disease‐specific measures are available for assessment of health‐related quality of life (HRQoL) in children, adolescents and adults with haemophilia,^16^, ^17^, ^18^ but HRQoL and health status outcomes data are limited for PwHA with inhibitors. Moreover, until recently,^19^, ^20^ most studies assessing HRQoL had been conducted as part of interventional clinical trials, primarily in PwHA without inhibitors.^21^, ^22^ Consequently, additional real‐world data (RWD) assessments are needed to determine HRQoL and overall health status in PwHA with inhibitors receiving routine clinical care.

An international non‐interventional study (NIS) was conducted to prospectively collect detailed RWD in PwHA, with and without inhibitors, treated according to local routine clinical practice. As long as they met respective eligibility criteria, participants from this cohort who were compliant with requirements of the NIS (ie, completed the Bleed and Medication Questionnaire on a regular basis) could start rolling over into the phase III HAVEN 1 study of emicizumab (HEMLIBRA^®^; F. Hoffmann‐La Roche, Basel, Switzerland), as soon as it was opened for enrolment at their treatment centre (ie, before week 25). Emicizumab is a subcutaneously administered recombinant humanized bispecific monoclonal antibody recently approved in several countries for prophylaxis in PwHA with inhibitors of all ages.^23^ Bleeding events and safety outcomes recently reported for PwHA with inhibitors aged ≥12 years in the NIS showed that bleeding rates remained high and compliance with activated prothrombin complex concentrate (aPCC) prophylaxis was suboptimal, with 40% of participants exhibiting low compliance to their prophylactic dosing frequency (administered prescribed number of doses <60% of study weeks).^24^ Participants did not treat ~40% of bleeds during the study, further supporting the need for more treatment options to decrease the burden of disease and treatment.^24^ The objective of this analysis was to characterize disease‐specific HRQoL, overall health status and the effect of bleeding on health status in PwHA with inhibitors aged ≥12 years.

## 2 MATERIALS AND METHODS

### 2.1 Study setting

The NIS design has previously been described.^24^ Briefly, this global, multicentre, prospective NIS (NCT02476942) enrolled PwHA into cohort A (PwHA with inhibitors aged ≥12 years), cohort B (PwHA with inhibitors aged <12 years) and cohort C (PwHA without inhibitors aged ≥12 years). Only the results from cohort A are reported here. Participants enrolled into this cohort from 26 May 2015‐29 February 2016.

This NIS was conducted at 33 centres in 12 countries (Australia, China, Costa Rica, Germany, Italy, Japan, Poland, Republic of Korea, South Africa, Spain, Taiwan and the United States). The data cut‐off was 31 March 2017 for the final analysis. The study was conducted in accordance with the International Conference on Harmonisation Guidelines for Good Clinical Practice, informed consent guidelines and the Declaration of Helsinki,^25^ and was approved by local ethics review groups. The protocol was developed by the sponsor, F. Hoffmann‐La Roche, Ltd.

### 2.2 Study participants

In cohort A, eligible participants with congenital haemophilia A (any severity) were aged ≥12 years; had a documented history of high‐titre FVIII inhibitors (≥5 Bethesda units/mL); documented episodic or prophylactic treatment for bleeds for ≥6 months prior to enrolment; and had experienced ≥6 bleeds on episodic treatment or ≥2 bleeds on prophylaxis in the prior 6 months. Participants receiving ITI with FVIII were ineligible.

### 2.3 Study design

The target participant number for each treatment type (episodic or prophylactic) enrolled into each cohort was estimated based on the approximate number of participants initially planned for the interventional emicizumab phase III pivotal study (NCT02622321).^26^ Participants remained in the NIS for ~6 months before being enrolled in the interventional study. The NIS was closed once all participants had switched to the interventional study or completed the NIS. Participants' usual care was continued throughout the study, with no protocol‐specified interventions.

### 2.4 Outcome measures

#### 2.4.1 HRQoL

HRQoL data were collected in adults using the Haemophilia Quality of Life Questionnaire for Adults (Haem‐A‐QoL), and in adolescents using the Haemophilia‐specific Quality of Life Questionnaire for Children Short Form (Haemo‐QoL SF).

The Haem‐A‐QoL is a validated measure designed to assess aspects of HRQoL in adult PwHA aged ≥18 years,^17^, ^27^, ^28^, ^29^, ^30^ consisting of 46 items in 10 domains (“Physical Health,” “Feelings,” “View of Yourself,” “Sports & Leisure,” “Work & School,” “Dealing With Haemophilia,” “Treatment,” “Future,” “Family Planning” and “Partnership & Sexuality”), which create a “Total” score when combined. Responses, based on participants' experience in the previous 4 weeks, could range from “never” (1) to “all of the time” (5) (5‐point Likert scale). In the “Sports & Leisure,” “Family Planning” and “Work & School” domains, a “not applicable” option was available. In order to score all responses in the same direction, “View of Yourself,” “Sports & Leisure,” “Work & School,” “Dealing with Haemophilia,” “Treatment” and “Future” domains were reverse‐scored. All scores were transformed to a 0‐100 scale, with higher scores reflecting greater impairment.

The Haemo‐QoL SF, a 35‐item measure designed to assess aspects of HRQoL in children and adolescents aged 8‐17 years, is composed of 9 domains (“Physical Health,” “Feelings,” “View of Yourself,” “Family,” “Friends,” “Other People,” “Sports & School,” “Dealing With Haemophilia,” and “Treatment”),^16^ which create a “Total” score when combined. Responses, based on participants' experience in the previous 4 weeks, could range from “never” (1) to “always” (5). The “View of Yourself,” “Friends,” “Sports & School,” and “Dealing with Haemophilia” domains were reverse‐scored. All scores were transformed to a 0‐100 scale, with higher scores reflecting greater impairment.

#### 2.4.2 Health status

Health status was assessed in all participants using the EuroQol 5‐Dimensions 5‐Level (EQ‐5D‐5L) questionnaire,^31^, ^32^ a reliable and valid tool for assessing health status in PwHA with and without inhibitors.^19^, ^33^ The five dimensions of the EQ‐5D‐5L assess “Mobility,” “Self‐care,” “Usual activities,” “Pain/discomfort” and “Anxiety/depression,” each with five levels of severity, ranging from “no problems” to “extreme problems” on the day of questionnaire completion.^32^, ^34^ The five dimensions were combined into an index utility score (IUS) using the UK “crosswalk” value set; scores range from −0.594 (extreme problems on all dimensions) to 1 (no problems on all dimensions).^35^ Participants rated their general health using the EQ visual analogue scale (EQ‐VAS), in which 0 and 100 are the worst and best imaginable health status, respectively. The EQ‐5D‐5L and EQ‐VAS were completed on regularly scheduled reporting days and on days when bleeding occurred.

#### 2.4.3 Absences from work or school

Every 4 weeks, participants were asked to report the number of missed work or school days during the previous 4 weeks, and the number of days they expected to attend. The proportion of missed days was calculated for each participant by dividing the number of missed days by the number of days they should have attended. The mean proportion of missed days for each group was calculated from the sum of the proportions for each participant, divided by the number of participants.

#### 2.4.4 Hospitalizations

Hospitalizations during the study period were recorded in the serious adverse events page of the electronic case report forms (eCRFs) by investigators every 4 weeks.

### 2.5 Data collection and analysis

Clinicians collected demographic data and medical histories from participants' medical records onto the eCRFs.

All other outcomes, except hospitalizations (described above), were recorded by participants using an electronic handheld device provided at enrolment. Participants completed all HRQoL and health status questionnaires on a 4‐weekly basis for ~6 months. Patients completed an additional EQ‐5D‐5L and EQ‐VAS on days when bleeding occurred.

Summary statistics for the HRQoL questionnaires and the EQ‐5D‐5L IUS and EQ‐VAS were calculated at each scheduled assessment; no statistical testing was performed. The analysis of IUS and EQ‐VAS when a bleed occurred (unscheduled assessments) versus when no bleed occurred (monthly scheduled assessments) included only participants with at least one scheduled and one unscheduled assessment.

The completion rates for Haem‐A‐QoL, Haemo‐QoL SF and scheduled EQ‐5D‐5L and EQ‐VAS were calculated by dividing the number of completed questionnaires by the number of questionnaires expected at each time point (determined from the number of eligible participants on study at the time). Completion rates for unscheduled EQ‐5D‐5L and EQ‐VAS were calculated as the number of unscheduled questionnaires completed, divided by number of bleeding days.

The proportion of days away from work/school in the past 4 weeks were reported separately, and were determined based on the expected number of days of attendance and absence, as documented by the participant.

All analyses were regarded as exploratory and descriptive; the study was not designed to make confirmatory claims.

## 3 RESULTS

### 3.1 Study population

Cohort A included 103 PwHA with inhibitors on episodic (n = 75) or prophylactic (n = 28) treatment with a median (range) age of 31 (12‐75) years (Table 1). The median (range) observation time/efficacy period was 25 (4‐70) weeks in the episodic group and 27 (8‐49) weeks in the prophylaxis group.^24^ Data are presented only through week 25 (day 1 ± 3 days) as the number of participants declined beyond this time due to rollover into HAVEN 1. Most participants (78/103 [76%]) reported receiving aPCC as either usual prophylaxis or for treatment of bleeding (Table 1).

**Table 1:** Participant demographics and treatment regimen

	Episodic (n = 75)	Prophylaxis (n = 28)	All (N = 103)
Sex, n (%)			
Male	74 (99)	28 (100)	102 (99)
Female^a^	1 (1)	0	1 (1)
Age			
Median, years (min, max)	33 (13, 75)	23 (12, 75)	31 (12, 75)
Age group, n (%)			
≥12 and < 18 y	8 (11)	9 (32)	17 (17)
≥18 y	67 (89)	19 (68)	86 (84)
Race, n (%)			
White	41 (55)	18 (64)	59 (57)
Asian	25 (33)	8 (29)	33 (32)
Black/African American	9 (12)	1 (4)	10 (10)
Multiple	0	1 (4)	1 (1)
Treatments reported by participants, n (%)			
aPCC	53 (71)	25 (89)	78 (76)
rFVIIa	35 (47)	11 (39)	46 (45)
Other^b^	8 (11)	5 (18)	13 (13)
Purpose of all reported treatments, n (%)			
Treatment for bleed	70 (93)	25 (89)	95 (92)
Usual prophylaxis	6 (8)	27 (96)	33 (32)
One‐time prophylaxis	35 (47)	13 (46)	48 (47)
Procedure/surgery	10 (13)	1 (4)	11 (11)
Purpose of aPCC treatment, n (%)			
Treatment for bleed	53 (71)	23 (82)	76 (74)
Usual prophylaxis	4 (5)	22 (79)	26 (25)
One‐time prophylaxis	24 (32)	11 (39)	35 (34)
Procedure/surgery	3 (4)	1 (4)	4 (4)

aPCC, activated prothrombin complex concentrate; max, maximum; min, minimum; rFVIIa, recombinant activated factor VII

a History of high‐titre inhibitors (indicative of severe disease), with 12 bleeds in the prior 6 months

b “Other” includes standard half‐life factor VIII (n = 3, 5 and 8 in the episodic, prophylaxis and all groups, respectively), intravenous 1‐deamino‐8‐D‐arginine vasopressin (n = 2; episodic group), cryoprecipitate (n = 2; episodic group), fresh frozen plasma/whole blood (n = 1; episodic group). All given during the study and in the presence of inhibitors.

Completion rates at the first assessment were 75% (61/81) for Haem‐A‐QoL and 88% (15/17) for Haemo‐QoL SF. Rates remained high for Haem‐A‐QoL (>81%) and Haemo‐QoL SF (>73%) through week 25. Completion rates for the EQ‐5D‐5L were comparable: 85% (88/103) at the first assessment and >88% through week 25.

### 3.2 HRQoL and health status

All Haem‐A‐QoL, Haemo‐QoL SF, EQ‐5D‐5L IUS and EQ‐VAS scores, starting with week 1, reflect the HRQoL and health status of participants treated according to local clinical practice; treatment was initiated before enrolment in the NIS and continued according to local standards thereafter.

#### 3.2.1 HRQoL in adults: Haem‐A‐QoL

**Figure 1:**
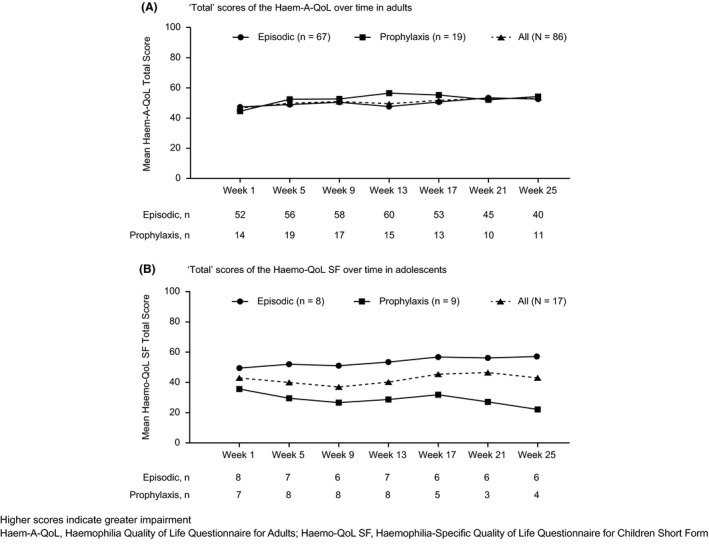
Haemophilia‐specific quality of life outcomes over time in PwHA with inhibitors receiving episodic or prophylactic treatment regimens

Among participants who completed the Haem‐A‐QoL at week 1, mean (standard deviation [SD]) Haem‐A‐QoL “Total” score was 47.3 (14.8) in the episodic group (n = 52) and 44.5 (16.7) in the prophylaxis group (n = 14). Mean Haem‐A‐QoL domain and total scores were generally comparable between episodic and prophylaxis groups and relatively consistent through week 25 (Table 2; Figure 1A). The domains with greatest impairments (ie, highest scores on a 0‐100 scale) in both groups at week 1 were “Sports & Leisure” and “Future”.

**Table 2:** Mean Haem‐A‐QoL domain and “Total” scores in adults at weeks 1 and 25

Haem‐A‐QoL domain scores, mean (SD)^a^	Episodic (n = 67)		Prophylaxis (n = 19)	
	Week 1 (n = 52)^b^	Week 25 (n = 40)^b^	Week 1 (n = 14)^b^	Week 25 (n = 11)^b^
Physical Health	53.1 (24.5)	58.1 (23.9)	48.5 (19.8)	64 (29.3)
Feelings	42.6 (23.6)	46.9 (26.1)	43.8 (25.4)	51.1 (34.2)
View of Yourself	52.8 (17.8)	57.9 (18.6)	50.1 (20.7)	58.2 (24.5)
Sports & Leisure	68.3 (24.3)	74.2 (19.9)	69.3 (25.9)	86.6 (10.9)
Work & School	43.5 (30.0)	52.6 (24.2)	41.7 (21.0)	50.0 (30.9)
Dealing With Haemophilia	31.5 (20.5)	39.0 (18.6)	31.4 (20.4)	22.4 (15.4)
Treatment	45.4 (18.4)	50.6 (18.3)	46.0 (18.9)	59.4 (24.8)
Future	53.4 (18.7)	55.5 (21.9)	51.5 (23.6)	64.0 (21.2)
Family Planning	37.8 (28.3)	48.6 (34.6)	25.9 (31.0)	50.8 (21.0)
Partnership & Sexuality	34.4 (32.6)	40.4 (32.1)	20.5 (29.2)	44.2 (42.1)
Total Score	47.3 (14.8)	52.6 (17.0)	44.5 (16.7)	54.3 (21.5)

Haem‐A‐QoL, haemophilia quality of life questionnaire for adults; SD, standard deviation

a Higher scores indicate greater impairment

b Values are for Total scores; n's were less for some of the domains

#### 3.2.2 HRQoL in adolescents: Haemo‐QoL SF

In adolescents who completed the initial Haemo‐QoL SF, mean (SD) Haemo‐QoL SF “Total” scores in the episodic (n = 8) and prophylaxis (n = 7) groups, respectively, were 49.5 (18.0) and 35.6 (10.9) at week 1, and 57.1 (21.0) and 22.1 (7.0) at week 25. The domains with greatest impairments (ie, highest scores on a 0‐100 scale) at week 1 were “Sports & School” and “Family” in the episodic group and “Sports & School” and “View of Yourself” in the prophylaxis group (Table 3). Although not tested for statistical significance, mean Haemo‐QoL SF domain and total scores suggest that adolescents receiving episodic treatment may have poorer HRQoL than those receiving prophylaxis (Table 3; Figure 1B).

**Table 3:** Mean Haemo‐QoL SF domain and “Total” scores in adolescents at weeks 1 and 25

Haemo‐QoL SF domain scores, mean (SD)^a^	Episodic (n = 8)		Prophylaxis (n = 9)	
	Week 1 (n = 8)^b^	Week 25 (n = 6)^b^	Week 1 (n = 7)^b^	Week 25 (n = 4)^b^
Physical Health	43.8 (12.1)	53.1 (31.3)	32.1 (18.9)	23.4 (11.8)
Feelings	53.9 (22.9)	60.4 (24.9)	25.9 (21.8)	12.5 (15.3)
View of Yourself	56.3 (31.7)	59.4 (33.3)	50.0 (26.0)	15.6 (12.0)
Family	60.9 (26.5)	65.6 (24.0)	24.1 (20.2)	14.1 (7.9)
Friends	34.4 (23.3)	44.4 (22.1)	42.9 (23.3)	39.6 (26.7)
Other People	36.7 (27.4)	43.8 (27.7)	27.7 (8.0)	17.2 (13.9)
Sports & School	71.1 (22.9)	81.3 (18.5)	52.7 (27.0)	29.7 (18.7)
Dealing With Haemophilia	40.6 (22.4)	46.9 (30.3)	39.3 (23.3)	18.8 (13.5)
Treatment	43.8 (21.1)	56.3 (16.3)	27.7 (16.9)	32.8 (25.7)
Total Score	49.5 (18.0)	57.1 (21.0)	35.6 (10.9)	22.1 (7.0)

Haemo‐QoL SF, haemophilia‐specific quality of life questionnaire for children short form; SD, standard deviation

a Higher scores indicate greater impairment

b Values are for total scores; n's were less for some of the domains

#### 3.2.3 Health status: EQ‐5D‐5L IUS and EQ‐VAS

**Figure 2:**
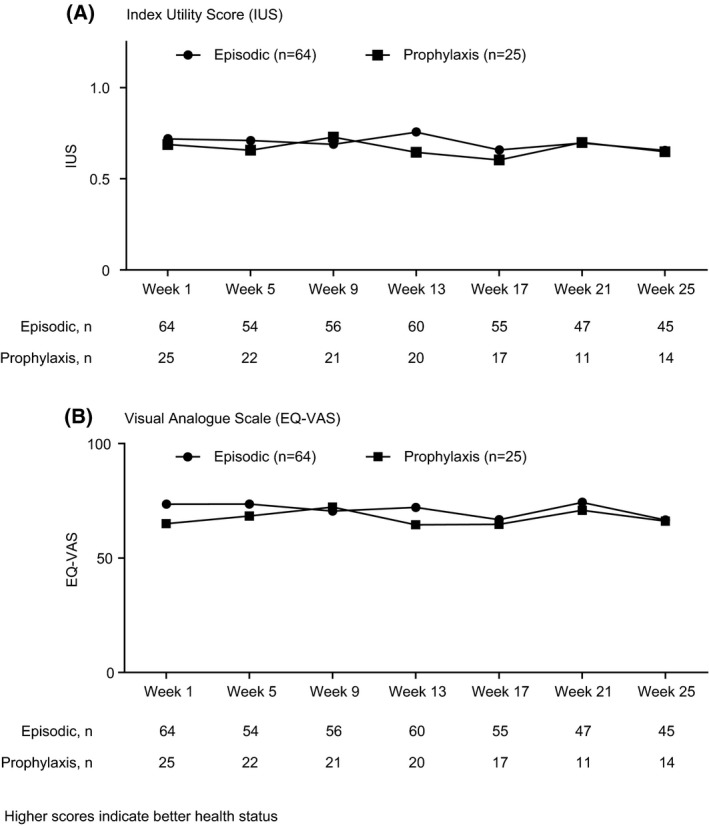
Health status according to the EuroQol 5‐Dimensions 5‐Levels over time in PwHA with inhibitors receiving episodic or prophylactic treatment regimens

**Figure 3:**
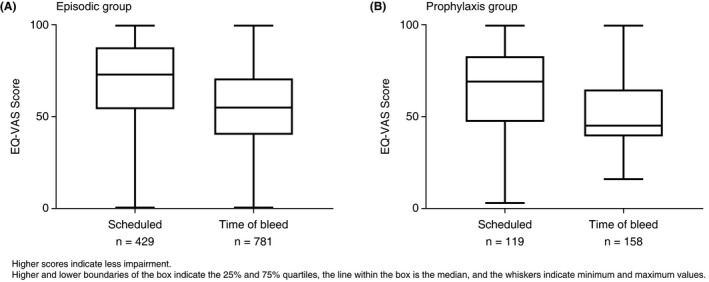
Effect of bleeding on health status. EuroQol visual analogue scale (EQ‐VAS) score at time of scheduled assessment and on days during which a bleed occurred

At week 1, in the episodic and prophylaxis groups, respectively, mean (SD) EQ‐5D‐5L IUS scores were 0.72 (0.23) and 0.69 (0.24), and mean (SD) EQ‐VAS scores were 73.5 (20.1) and 65.0 (25.0). Although mean baseline EQ‐5D‐5L IUS and EQ‐VAS scores were higher in the episodic group than the prophylaxis group, there was no consistent difference between the groups and no systematic change in either value during the study (Figure 2). Bleeding had a marked impact on IUS and EQ‐VAS scores. Mean IUS scores were higher in the episodic and prophylaxis groups, respectively, at regularly scheduled assessments (0.68; 0.62) versus bleeding days (0.51; 0.48). Similarly, mean EQ‐VAS scores in the episodic and prophylaxis groups, respectively, were higher during regularly scheduled assessments (69.6; 64.5) than on bleeding days (54.7; 51.7) (Figure 3).

### 3.3 Absence from work/school and hospitalizations

Assessment of work absenteeism included 44 of 75 (59%) participants in the episodic group and 12 of 28 (43%) participants in the prophylaxis group. One participant in each treatment group did not complete the questionnaire, while the remaining participants were not working (episodic, n = 30; prophylaxis, n = 15). The mean (95% confidence interval [CI]) proportion of days away from work for all patients was 16% (10%‐22%) and was comparable between groups (episodic: 16% [9%‐22%]; prophylaxis: 18% [3%‐34%], respectively).

Assessment of school absenteeism included 35 of 75 (47%) participants in the episodic group and 16 of 28 (57%) participants treated with prophylaxis. One participant in each treatment group did not complete the questionnaire, while the remaining participants were not in school (episodic, n = 39; prophylaxis, n = 11). The mean (95% CI) proportion of days away from school for all patients was 23% (14%‐32%) and was comparable between groups (episodic: 21% [12%‐30%]; prophylaxis 29% [7%‐50%]).

The mean (SD) number of days hospitalized was comparable between groups (episodic: 2.7 [8.9]; prophylactic: 4.5 [8.5]), and the median number of days hospitalized in each group was zero (most participants were not hospitalized). The number of days hospitalized ranged from 0 to 50 in the episodic group and from 0 to 26 in the prophylaxis group.

## 4 DISCUSSION

This prospective NIS collected detailed RWD on bleeding and health‐related outcomes in PwHA with inhibitors aged ≥12 years (cohort A) treated according to local routine clinical practice. The results presented here show these individuals experienced impairments (indicated by higher scores in the Haem‐A‐QoL and Haemo‐QoL SF, respectively) in HRQoL despite standard treatment, with mean scores in the majority of HRQoL domains in adults and adolescents indicative of impairments reported, on average, to occur “sometimes” to “often.”

These health‐related outcomes may result from a combination of poor bleed control and treatment burden. Bleeding outcomes for this NIS cohort have been published,^24^ and showed that annualized bleeding rates (95% CI) for all bleeds and treated bleeds, respectively, were high with both episodic (33 [27‐39]; 19 [15‐23]) and prophylactic (25 [18‐34]; 15 [11‐21]) treatment, and that most bleeds occurred in joints. Participants did not treat ~40% of bleeds, and compliance with prophylactic treatment was low, likely reflecting the high burden associated with standard therapies.^24^

In adults, mean Haem‐A‐QoL scores were similar at each analysis time point. Mean Haemo‐QoL SF scores among adolescents appeared to vary over time, but this should be interpreted with caution given the small number of adolescent participants. Because participants remained on their usual regimen, substantial changes in HRQoL and health status were not expected during the NIS. Haem‐A‐QoL and Haemo‐QoL SF total and domain scores were comparable with other reports for PwHA with inhibitors treated with standard of care.^12^, ^21^ Among all participants in cohort A, mean total HRQoL scores over time were higher, and impairment therefore greater, in adults than adolescents, as previously shown by Dekoven, et al.^12^

Adult PwHA with inhibitors reported greatest impairments (highest scores) in the “Sports & Leisure” domain of the Haem‐A‐QoL, reflecting reports that they often had to refrain from participating in these activities. The high scores for the “Physical Health” and “Sports & Leisure” domains, as well as the high “Total” score, are not unexpected given the high bleeding rates reported for this cohort. Similarly, on the Haemo‐QoL SF (adolescents), the “Sports & School” domain showed greatest impairment.

There was no systematic change in EQ‐5D‐5L IUS or EQ‐VAS throughout the study. At week 1, mean EQ‐VAS was lower in the prophylaxis than the episodic group, but the scores were comparable at most other time points. Mean EQ‐5D‐5L IUS scores in PwHA with inhibitors in the present study (0.72 and 0.69 in episodic and prophylaxis groups, respectively) were lower than those reported for PwHA with inhibitors in the US Centers for Disease Control and Prevention Universal Data Collection system (0.75),^36^ and those reported in the Pain, Functional Impairment, and Quality of Life study (P‐FiQ) (0.78).^6^ The results in P‐FiQ, however, may have been due to the low percentage (33/381; 8.7%) of subjects with inhibitors enrolled. Consistent with previous studies,^37^ health status as measured by the EQ‐5D‐5L IUS and EQ‐VAS in the present study was measurably better in the absence of bleeding than on bleeding days, and this effect was comparable between episodic and prophylaxis groups.

The impact of haemophilia A and associated bleeding events on daily activities in PwHA with inhibitors is not well documented. In the NIS cohort A, the overall mean proportions (95% CI) of missed work or school days were 16% (10%‐22%) and 23% (14%‐32%), respectively, and were comparable in the episodic and prophylaxis groups. In the Dosing Observation Study in Haemophilia, PwHA with inhibitors receiving BPAs and their caregivers missed many full days (13.5% and 9.3%, respectively) from work/school.^38^ Overall, most participants in cohort A were not hospitalized, and the average duration of hospitalization was 3.2 days (median, 0 days).

The limitations of the study included the small number of adolescent participants, potential selectivity of the sample, and participant attrition rate. The latter limitation is reflected in the fact that a number of data sets at each time point were incomplete, particularly later in the study. There are many possible reasons for this, including participant rollover into the phase III HAVEN 1 emicizumab clinical trial.

## 5 CONCLUSION

These RWD from cohort A of the NIS demonstrate that adolescent and adult PwHA with inhibitors experience substantially impaired health‐related outcomes while receiving standard care, revealing an unmet need for more effective treatment options.

## DISCLOSURES

J. Mahlangu has received research grants from Bayer, Biogen, BioMarin, CSL Behring, Novo Nordisk, Sobi, Roche and Unique Pharmaceuticals; been a member of scientific advisory committees for Amgen, Bayer, Biotest, Biogen, Baxalta, CSL Behring, Catalyst Biosciences, Novo Nordisk, Roche and Spark Therapeutics; and been a speaker bureau member for Alnylam, Bayer, Biotest, Biogen, Novo Nordisk, Pfizer, Sobi, Shire, Roche, ISTH and WFH. J. Oldenburg has received reimbursement for attending symposia/congresses and/or honoraria for speaking/consulting and/or funds for research from Bayer, Biogen, Biotest, Chugai Pharmaceutical Co., CSL Behring, Grifols, Novo Nordisk, Octapharma, Pfizer, Roche, Shire and Sobi. M.U. Callaghan has been a paid consultant and speaker for Roche/Genentech; a paid speaker for Bayer, Novo Nordisk and Shire; has been a paid consultant for Bayer, Bioverativ, Grifols, HEMA Biologics, Pfizer and Shire; and owns stock in Alnylam. M. Shima is a board member of the Feiba and Advate Safety Board in Japan organized by Baxalta; has received payment for consultancy meetings with Baxalta, Bayer, Biogen, Chugai Pharmaceutical Co., CSL Behring, Kaketsuken, Novo Nordisk and Pfizer; and has received unrestricted grants supporting research from Baxalta, Bayer, Chugai Pharmaceutical Co., CSL Behring, Kaketsuken, Novo Nordisk and Pfizer. M. E. Mancuso has acted as a speaker for Baxalta/Shire, Bayer, Biotest, CSL Behring, Novo Nordisk, Octapharma, Pfizer and Roche; and been a paid consultant for Baxalta/Shire, Bayer, CSL Behring, Kedrion, Novo Nordisk, Pfizer and Roche. P. Trask is an employee of Genentech, Inc. M. Recht has received grant/research support from Bioverativ, Genentech, Novo Nordisk and Shire, and is a paid consultant for Bioverativ, CSL Behring, Genentech, Kedrion, Novo Nordisk, Pfizer, Shire and uniQure. C. Garcia has received reimbursement for attending symposia for Janssen and Novo Nordisk; and received speaking fees from Janssen, Novartis, Novo Nordisk, Pfizer and Roche. R. Yang has nothing to disclose. M. Lehle, H. Macharia and E. Asikanius are employees of F. Hoffmann‐La Roche, Ltd. G. G. Levy is an employee of Genentech, Inc and owns Roche stock. Kruse‐Jarres has acted as a paid consultant to Grifols, Novo Nordisk, Pfizer, Roche and Shire; and has received research funding from CSL Behring, Pfizer and Roche. S. von Mackensen is a consultant for Roche.
